# ApoA-I/A-II-HDL positively associates with apoB-lipoproteins as a potential atherogenic indicator

**DOI:** 10.1186/s12944-017-0619-y

**Published:** 2017-11-29

**Authors:** Toshimi Kido, Kazuo Kondo, Hideaki Kurata, Yoko Fujiwara, Takeyoshi Urata, Hiroshige Itakura, Shinji Yokoyama

**Affiliations:** 1 Institute for Human Life Innovation, Ochanomizu University, Tokyo, 112-8610 Japan; 2 Department of Food and Nutritional Science, Toyo University, Itakura-machi, Gunma 374-0193 Japan; 3 Division of Diabetes, Metabolism and Endocrinology, Department of Internal Medicine, The Jikei University School of Medicine, Tokyo, 105-8461 Japan; 4 Department of Diabetes, Metabolism and Endocrinology, Showa University School of Medicine, Tokyo, 142-8555 Japan; 5 Shinagawa East One Medical Clinic, Tokyo, 108-0075 Japan; 6 Nutritional Health Science Research Center, Chubu University, Kasugai, Aichi 487-8501 Japan

**Keywords:** HDL, apoA-I, apoA-II, apoB, CETP

## Abstract

**Background:**

We recently reported distinct nature of high-density lipoproteins (HDL) subgroup particles with apolipoprotein (apo) A-I but not apoA-II (LpAI) and HDL having both (LpAI:AII) based on the data from 314 Japanese. While plasma HDL level almost exclusively depends on concentration of LpAI having 3 to 4 apoA-I molecules, LpAI:AII appeared with almost constant concentration regardless of plasma HDL levels having stable structure with two apoA-I and one disulfide-dimeric apoA-II molecules (Sci. Rep. 6; 31,532, 2016). The aim of this study is further characterization of LpAI:AII with respect to its role in atherogenesis.

**Methods:**

Association of LpAI, LpAI:AII and other HDL parameters with apoB-lipoprotein parameters was analyzed among the cohort data above.

**Results:**

ApoA-I in LpAI negatively correlated with the apoB-lipoprotein parameters such as apoB, triglyceride, nonHDL-cholesterol, and nonHDL-cholesterol + triglyceride, which are apparently reflected in the relations of the total HDL parameters to apoB-lipoproteins. In contrast, apoA-I in LpAI:AII and apoA-II positively correlated to the apoB-lipoprotein parameters even within their small range of variation. These relationships are independent of sex, but may slightly be influenced by the activity-related CETP mutations.

**Conclusions:**

The study suggested that LpAI:AII is an atherogenic indicator rather than antiatherogenic. These sub-fractions of HDL are to be evaluated separately for estimating atherogenic risk of the patients.

## Background

High-density lipoprotein (HDL) plays a central role in cholesterol transport from peripheral tissues to the liver where it is converted to bile acids for enterohepatic circulation and excretion, as an essential part of its catabolism. HDL thus appears as an apparent negative risk factor for atherosclerotic diseases and is believed to act against atherogenesis. However, many attempts to increase plasma HDL failed to prevent vascular diseases, in contrast to their evident decrease by reduction of a positive risk factor plasma low-density lipoprotein (LDL). Complexity of the HDL pathway that involves so many steps and factors may make it difficult to optimize its manipulation for prevention of atherosclerosis.

HDL is a group of small lipid-protein particles composed of hydrophobic core of cholesteryl ester (CE) with a small amount of triglyceride (TG) and of surface layer of phospholipid, unesterified cholesterol and apolipoproteins that bind to lipid by amphiphilic helices [1]. The particles are physicochemically unstable and metabolically active so that their structure is in a dynamic equilibrium rather than stable construction. HDL is therefore found with subsets of the particles based on parameters such as density, size, or apolipoprotein composition. However, these HDL subgroups have not clearly been defined for their functions in cholesterol transport or roles in atherogenesis. We recently reported that traditional two major subsets of human HDL classified by apolipoprotein composition, the particles with apolipoproteins A-I (apoA-I) but not A-II (apoA-II) (LpAI) and those having both (LpAI:AII), are distinct from each other with respect to structural stability and apparent metabolic fate by analyzing 314 plasma samples from a Japanese cohort study [2]. The former particles are of variational structure and concentration being composed of 3 to 4 apoA-I molecules and determine total plasma HDL level. On the other hand, the latter particles appear with stable structure containing two apoA-I and one disulfide-dimeric apoA-II molecules and little-variable plasma concentration regardless of total HDL level. Thus, these two HDL subsets seem profoundly different in structure and metabolism. It is therefore important to clarify their functional differences to understand cholesterol transport by HDL which is overall considered as antiatherogenic, and to design the strategy for prevention of atherosclerosis by manipulating HDL metabolism. We here intended to carry out further bioinformatic analysis of these HDL subsets to examine their differential roles in atherogenesis.

## Methods

Fasting blood plasma samples were collected from the subjects randomly selected from those who visited Omiya City Clinic for regular health check-up being blinded for background information, 177 males and 137 females, as reported in detail in the previous publication [2].

CETP genotype was determined for intron 14 (1452G–A) and exon 15 (D442G) mutations which are known to explain most of genetic CETP activity deficiency in Japanese [3]. One male homozygote and 20 male and 17 female heterozygotes were identified among the subjects above as previously reported [2].

Total cholesterol (TC), TG, and HDL-cholesterol (HDL-C) levels in plasma were determined by enzymatic methods by using commercially available assay kits (SEIKEN T-CHO(S), SEIKEN FG-TG(II), SEIKEN HDL-CHO, respectively, DENKA SEIKEN, Ltd., Tokyo) in a biochemical analyzer TBA-60R (TOSHIBA MEDICAL SYSTEMS CORPORATION, Tochigi, Japan). ApoA-I, apoA-II and apolipoprotein B (apoB) were determined by using commercial immunoturbidimetry assay systems (apoA-I auto•2, apoA-II auto•2, apoB auto•2 Daiichi Pure Chemicals Co., Ltd., Tokyo) [2]. All procedures of measurement above mentioned were done automatically with a biochemical analyzer COBAS MIRA (Roche Diagnostics Corporation, Indianapolis, USA). LpAI was evaluated by measuring apoA-I concentration in the supernatant after immuneprecipitation of LpAI:AII with anti apoA-II antibody, and LpAI:AII was estimated by subtracting this value from total apoA-I as well as by plasma apoA-II concentration [2]. ApoA-I in LpAI was thus determined as apoA-II-unassociated apoA-I by using the HYDRAGEL LPAI PARTICLES Kit (Sebia, Issy-les-Moulineaux, France) by electroimmunodiffusion technique [4] as previously reported [2], being validated by a method of combination of immuneprecipitation of LpAI:AII with anti-apoA-II antibody and turbidimetric immunoassay with anti apoA-I antibody [2].

Correlation between two sets of the data was analyzed by simple linear or logarithmic linear regression performed along with evaluation of significance of correlation by using GraphPad Prism 5 for Windows (GraphPad Software, San Diego, CA).

## Results and discussion

**Fig. 1:**
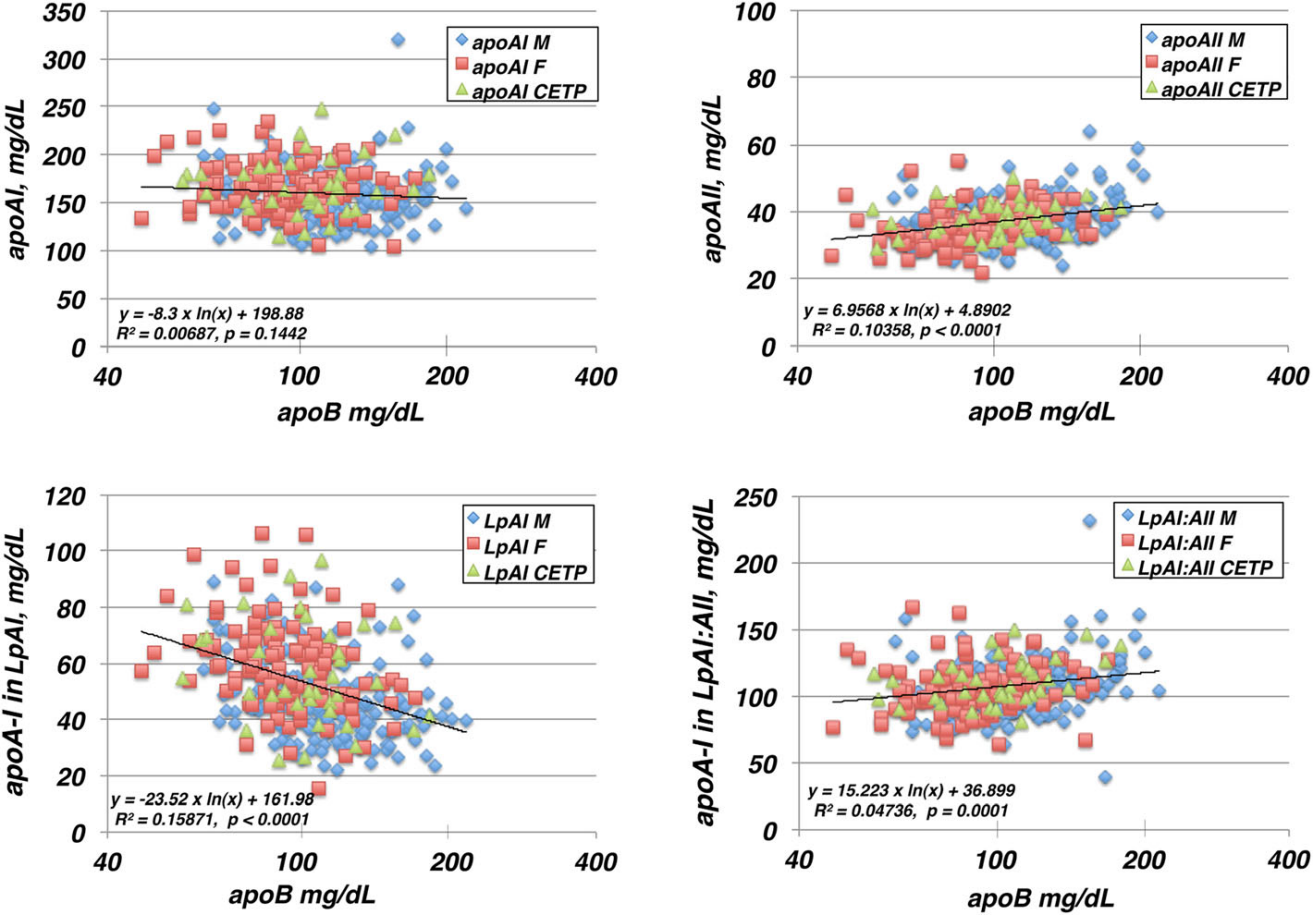
Correlation of HDL apolipoprotein parameters, apoA-I, apoA-II, apoA-I in LpAI and apoA-I in LpAI:AII to apoB, as displayed for male (M, *n* = 155) and female (F, *n* = 120) of the subjects with normal CETP gene and those with CETP mutations (CETP, *n* = 37). ApoB is shown as logarithmic scale in the graphs and the lines represent logarithmic liner regression of the total samples

Of the 314 cohort subjects, one male homozygote of CETP mutation (D442G) and one CETP normal male with very high TG (1438 mg/dl) were excluded from the analysis hereafter. Relationship of HDL apolipoprotein markers including those for its subfractions, total apoA-I, apoA-I in LpAI and LpAI:AII and apoA-II, to apoB, an apolipoprotein parameter to indicate particle number of LDL and very low density lipoprotein (apoB- lipoproteins), is displayed in Fig. 1. No significant correlation was found between total apoA-I and apoB. However, apoA-I in LpAI, measured as apoA-II-unassociated apoA-I and a determinant of total apoA-I, showed strong inverse relationship to apoB, as 50% decrease over an increase of apoB by a factor of 4. In contrast, apoA-II was found increased at most 25% with significant positive correlation to apoB. Accordingly, apoA-I in LpAI:AII also showed positive correlation to apoB to a similar extent. Metabolic nature of LpAI and LpAI:AII thus appear distinct from each other demonstrated by their opposite correlation to apoB-lipoprotein concentration.

Table 1 lists correlation of these HDL subfraction markers as well as total HDL markers such as HDL-C to other indicators of apoB-lipoprotein, TG, nonHDL-C (TC – HDL-C) and TG + nonHDL-C, in addition to apoB. The relationships of the HDL markers to the apoB-lipoprotein indicators were found mostly similar to what were found with those to apoB. Total apoA-I was however found in significant negative correlation only with TG and TG + nonHDL-C. On the other hand, HDL-C was in strong inverse correlation with all of the apoB-lipoprotein indicators. The findings were essentially similar among the subgroups of males and females of the CETP-normal and the CETP mutants, except that the CETP mutants appeared with reduced significance (Fig. 1 and Table 2).

**Table 1:** Correlation of HDL parameters to apoB-lipoprotein indicators for the total cohort excluding one CETP mutation homozygote and one CETP normal male with TG 1438 mg/dL (*n* = 312). ApoB-lipoprotein parameters are treated in a logarithmic scale except for nonHDL-C

y	x	Regression	r^2^	p
HDL-C	apoB	y = −24.13 ln(x) + 169.01	0.21634	< 0.0001
	TG	y = −17.07 ln(x) + 136.57	0.31917	< 0.0001
	nonHDL-C	y = −0.1536 x + 77.838	0.11965	< 0.0001
	TG + nonHDL-C	y = −24.5 ln(x) + 191.47	0.28738	< 0.0001
apoA-I	apoB	y = −8.3 ln(x) + 198.88	0.00687	0.1442
	TG	y = −7.434 ln(x) + 195	0.01625	0.0243
	nonHDL-C	y = −0.0514 × + 167.33	0.0036	0.2906
	TG + nonHDL-C	y = −10.59 ln(x) + 218.45	0.0144	0.0341
apoA-II	apoB	y = 6.9568 ln(x) + 4.8902	0.10358	< 0.0001
	TG	y = 3.5411 ln(x) + 20.668	0.07915	< 0.0001
	nonHDL-C	y = 0.0522 × + 30.102	0.07973	< 0.0001
	TG + nonHDL-C	y = 5.8489 ln(x) + 5.0808	0.09432	< 0.0001
apoA-I in LpAI	apoB	y = −23.52 ln(x) + 161.98	0.15871	< 0.0001
	TG	y = −15.1 ln(x) + 123.19	0.19289	< 0.0001
	nonHDL-C	y = −0.1628 × + 74.852	0.10373	< 0.0001
	TG + nonHDL-C	y = −22.9 ln(x) + 178.46	0.19376	< 0.0001
apoA-I in LpAI:AII	apoB	y = 15.223 ln(x) + 36.899	0.04736	0.0001
	TG	y = 7.6666 ln(x) + 71.809	0.03542	0.0008
	nonHDL-C	y = 0.1113 × + 92.476	0.03456	0.0010
	TG + nonHDL-C	y = 12.312 ln(x) + 39.988	0.0399	0.0004

**Table 2:** Correlation of HDL parameters to apoB for subsets of samples of normal male (M, *n* = 155) and female (F, *n* = 120), and of CETP mutants (CETP, *n* = 37). ApoB is treated in a logarithmic scale

y	x	Regression	r^2^	p
HDL-C	apoB (M)	y = −20.7 ln(x) + 148.97	0.19402	< 0.0001
	apoB(F)	y = −18.25 ln(x) + 145.65	0.12204	< 0.0001
	apoB(CETP)	y = −18.11 ln(x) + 146.74	0.09913	0.0577
apoA-I	apoB (M)	y = 3.2611 ln(x) + 138.53	0.00095	0.7039
	apoB(F)	y = −12.28 ln(x) + 221.9	0.01573	0.1723
	apoB(CETP)	y = 7.8031 ln(x) + 132.09	0.00536	0.6668
apoA-II	apoB(M)	y = 6.9663 ln(x) + 4.9046	0.08481	0.0002
	apoB(F)	y = 6.9288 ln(x) + 4.6302	0.09889	0.0005
	apoB(CETP)	y = 5.0424 ln(x) + 14.743	0.07704	0.0963
apoA-I in LpAI	apoB(M)	y = −17.01 ln(x) + 127.03	0.10534	< 0.0001
	apoB(F)	y = −21 ln(x) + 154.85	0.11063	0.0002
	apoB(CETP)	y = −15.75 ln(x) + 129.42	0.05717	0.1541
apoA-I in LpAI:AII	apoB(M)	y = 20.269 ln(x) + 11.498	0.06792	0.0011
	apoB(F)	y = 8.7152 ln(x) + 67.055	0.01481	0.1855
	apoB(CETP)	y = 23.554 ln(x) + 2.6636	0.14426	0.0204

The overall results showed a clear difference between the HDL particles without apoA-II (LpAI) and those with apoA-II (LpAI:AII) with respect to their relations to apoB-lipoprotein metabolism. The findings indicate that LpAI:AII is apparently an atheorgenic index, being functionally distinct from an apparent antiatherogenic maker LpAI.

Classification of HDL into the particles with and without apoA-II (LpAI:AII and LpAI) is one of the classical differentiations of human HDL subfractions [5–10] though functional and structural differences of these particles have not fully and clearly been distinguished. Nevertheless, LpAI is said to be more responsible for anti-atherogenic nature of HDL because increase of HDL is mostly in this subfraction [11–13] or it seems biologically more active in such reactions as cell cholesterol removal [14], endothelial protection [15] and selective CE uptake by scavenger receptor BI [16]. Clinical relevance of such hypotheses has, however, not been fully confirmed [4, 17–19]. Views and opinions on metabolic stability of these HDL subfractions are also controversial [11, 20–22].

Plasma lipoproteins compose the two major cholesterol transport systems, apoB-lipoproteins for normal direction from the liver to the somatic cells and HDL for reverse transport from the peripheral cells to the liver, which are essentially under independent regulation as to gene expression related to each pathway. However, CETP shunts these two pathways by mediating net transfer of CE generated in HDL to apoB-lipoproteins creating a by pass for the reverse transport of cholesterol to the liver via the hepatic LDL receptor. This net move of CE from HDL to apoB-lipoproteins is generated by equimolar exchange of CE in HDL and TG in apoB-lipoprotein, so that increase of plasma TG reduces HDL-C [23, 24]. This may cause not only decrease of HDL-C but also change in other HDL indicators including its diameter due to subsequent remodeling of the HDL particles [23, 24]. The present data indeed showed strong inverse relationship of HDL-C to TG (Tables 1 and 2).

We recently reported that distribution of apoA-II among HDLs to form LpAI:AII HDL is consistent with a statistical probability model [2] based on the assumption of higher affinity of apoA-II than apoA-I to lipid particles [25, 26]. LpAI:AII particles are structurally stable having one disulfide-dimer apoA-II and two apoA-I molecules and its plasma concentration is largely constant regardless of total HDL concentration [2]. Therefore, variation of HDL concentration is almost exclusively dependent on LpAI. The current findings further demonstrated that inverse relationship to apoB-lipoproteins is found in fact only with LpAI. In contrast, LpAI:AII positively correlates with apoB-lipoprotein indicators. We thus conclude that LpAI and LpAI:AII are metabolically distinct. LpAI:AII is of stable structure and largely constant concentration, and shows significant but small increase along with the increase of apoB-lipoproteins. This relationship may be modulated by plasma CETP activity as its significance seems slightly reduced among heterozygous CETP-mutant subjects due to their reduced CE/TG exchange rate influencing this steady state [24].

It is clear that apoA-II is the determinant to generate LpAI:AII. Our knowledge on function of apoA-II is however very limited. In addition to its higher affinity to HDL surface [25, 26], apoA-II was shown to enhance CETP reaction so much as apoA-I [27–29], but demonstrated as a much poorer activator of LCAT than apoA-I [30, 31]. It is unclear whether these differences interpret generation of unique LpAI:AII. LpAI:AII may not directly be resistant to CETP reaction but its structural stability may act against remodeling caused by CETP reaction. LpAI, in contrast, seems more variant to determine total HDL concentration and susceptible to CETP-triggered remodeling to appear in inverse correlation with apoB-lipoproteins. Otherwise, the influence by CETP mutation could merely be caused by relatively small sample size.

## Conclusions

In addition to our previous demonstration that LpAI and LpAI:AII are distinct from each other structurally and metabolically [2], we here demonstrated positive relationship of LpAI:AII with apoB lipoproteins in contrast to the negative association of LpAI. Accordingly, we predict that LpAI:AII is an atherogenic parameter in contrast to LpAI or other HDL parameters as antiatherogenic indicators. Thus, LpAI and LpAI:AII HDL are functionally different, with respect to cholesterol transport and accordingly antiatherogenic activity. This is important information to understand mechanism of antiathrogeneity of HDL and to construct strategy for prevention/cure of atherosclerosis by raising HDL.

## Abbreviations

apoA-I: Apolipoprotein A-I
apoA-II: Apolipoprotein A-II
apoB: Apolipoprotein B
CE: Cholesteryl ester
CETP: Cholesteryl ester transfer protein
HDL: High density lipoprotein
HDL-C: HDL-cholesterol
LDL: Low density lipoprotein
LpAI: HDL containing apoA-I but no apoA-II
LpAI:AII: HDL containing both apoA-I and apoA-II
nonHDL-C: TC – HDL-C
TC: Total cholesterol
TG: Triglyceride
